# Effects of Amazake Produced with Different *Aspergillus* on Gut Barrier and Microbiota

**DOI:** 10.3390/foods12132568

**Published:** 2023-06-30

**Authors:** Hironobu Nakano, Sho Setoguchi, Kuniaki Kawano, Hiroshi Miyagawa, Kozue Sakao, De-Xing Hou

**Affiliations:** 1 The United Graduate School of Agricultural Sciences, Kagoshima University, 1-21-24 Korimoto, Kagoshima 890-0065, Japan; k8108910@kadai.jp (H.N.); sakaok24@chem.agri.kagoshima-u.ac.jp (K.S.); 2 Kirishima Shuzo Co., Ltd., 4-28-1 Shimokawahigashi, Miyazaki 885-8588, Japank-kawano@kirishima.co.jp (K.K.); hiroshi-miyagawa@kirishima.co.jp (H.M.); 3 Faculty of Agriculture, Kagoshima University, 1-21-24 Korimoto, Kagoshima 890-0065, Japan

**Keywords:** fermented food, Amazake, *Aspergillus*, gut barrier, gut microbiome

## Abstract

Inflammatory bowel disease (IBD) is a chronic inflammatory disease of the gastrointestinal tract. To explore the preventive effects of dietary foods on IBD, we evaluated the effects of the traditional Japanese fermented beverage “Amazake” on gut barrier function in this study. Black koji Amazake (BA) derived from *Aspergillus luchuensis* MEM-C strain and yellow koji Amazake (YA) derived from *Aspergillus oryzae* were made in this study, and their nutrients were analyzed. Mice with mild gut barrier dysfunction induced by Western diet were administered with 10% of each Amazake for two months. Mice gut microbiota were analyzed by 16S rRNA gene sequencing. BA contained a higher amount of isomaltooligosaccharides, citric acid, and ferulic acid than YA. The animal data revealed that BA significantly induced the expressions of antioxidant factors and enzymes such as NF-E2-related factor 2 (Nfr2), heme oxygenase 1 (HO1), and superoxide dismutase-2 (SOD-2). The gut barrier protein, occludin, and fecal immunoglobulin A (IgA) were also significantly enhanced by BA. Furthermore, the levels of serum endotoxin and hepatic monocyte chemotactic protein-1 (MCP-1) were decreased in both the BA and YA groups. In gut microbiota, *Lachnospiraceae* was increased by BA while *Akkermansia muciniphilia* was increased by YA. Black koji Amazake contained a higher amount of isomaltooligosaccharides, citric acid, and ferulic acid than yellow koji Amazake and contributed to protecting gut barrier function to reduce endotoxin intrusion and inflammation.

## 1. Introduction

Inflammatory bowel disease (IBD) is a chronic inflammatory disease of the gastrointestinal tract, including Crohn’s disease and ulcerative colitis (UC), which have become important health problems. IBD’s prevalence was 0.059% worldwide (2019) [1] and 0.81% in the U.K. (2022) [2]. The direct causes and pathomechanisms of IBD are unclear. It is considered that the causes of IBD include genetic factors, environmental factors such as diet and smoking, immunological factors such as decreased regulatory T cells and increased T helper 17 cells, and impaired intestinal barrier function [3]. Conventional therapies for IBD include 5-aminosalicylic acid (5-ASA), corticosteroids, and thiopurine immunomodulators. Biologic agents such as anti-tumor necrosis factor-α (TNF-α) drugs, Janus kinase (JAK) inhibitors, and anti-α4β7 integrin antibodies have recently been developed [4]. Multiple studies have reported the effects of these therapeutics on gut tight-junction structures. Treatment with 5-ASA, anti-TNF-α, and JAK inhibitors restored gut tight-junction proteins such as occludin and zonula occludens-1 (ZO-1) in mice gut inflammation induced by dextran sodium sulfate (DSS) [5,6]. On the other hand, corticosteroids, a type of anti-inflammatory agents, decreased TNF-α as t but also had side effects such as increasing intestinal epithelial permeability [7]. Unfortunately, the prolonged use of these treatments is limited because they have serious side effects, including gastrointestinal problems such as diarrhea, abdominal pain, anemia, hepatotoxicity, and nephrotoxicity [8]. Based on this background, research on the treatment/prevention of IBD using probiotics/prebiotics and polyphenols has been conducted [8]. Various models of enteritis in animal experiments have been developed and broadly classified into drug models (e.g., DSS, oxazolone, and trinitrobenzene sulfonic acid were used as inducers) to mimic acute inflammation, and dietary models (e.g., high-fat diet) to mimic chronic inflammation, which caused a decrease in Paneth cell area, goblet cell count, antimicrobial peptides, mucin, and intestinal permeability as the expression of occludin [9].

Amazake is a traditional fermented rice beverage and has been consumed in Japan for over 1000 years, as first mentioned in the second-oldest book in Japan, Nihon Shoki (Chronicles of Japan). Koji Amazake is produced from steamed rice and water with *Aspergillus*. Amylase and protease from *Aspergillus* can break down rice into about 300 components, including isomaltooligosaccharides (IMOs), free amino acids, organic acids, and vitamins [10]. So far, Amazake has been reported to increase leukocytes and neutrophils in liver cirrhosis patients [11], improve skin properties in humans [12], suppress body weight (BW) and serum triglycerides in obese mouse models [13], and have 2,2-diphenyl-1-picrylhydrazyl radical scavenging ability [14]. To our knowledge, there are no studies about the effects of Amazake on the gut barrier function and microbiome. Interestingly, the mixture of sake cake and rice koji, ingredients of Amazake, increased the amount of fecal mucin, an index of gut barrier function in mouse models [15]. The Amazake ingredients are dependent on the kind of koji used. In this study, we produced two kinds of Amazake using different types of koji and then compared their ingredients. Finally, the effects of the two Amazake on gut barrier function and microbiota were investigated in a Western-diet-induced gut disturbance mouse model.

## 2. Materials and Methods

### 2.1. Chemicals and Reagents

Japanese white rice was purchased from Matsushita rice-cleaning mill (Miyazaki, Japan). Yellow rice koji was purchased from Akita Konno Co., Ltd. (Akita, Japan). Lard and cellulose were purchased from Sigma-Aldrich Co., LLC. (Tokyo, Japan). Soybean oil, cholesterol, choline bitartrate, and methionine were purchased from Nacalai Tesque, Inc. (Kyoto, Japan). AIN-93G mineral mix and AIN-93G vitamin mix were purchased from Oriental Yeast Co., Ltd. (Tokyo, Japan). Corn starch was purchased from Sanwa Starch Co., Ltd. (Nara, Japan). Edible acid casein 30–60 Mesh was purchased from Meggle (Wasserburg am Inn., Germany). Sucrose was purchased from Hayashi Pure Chemical Ind., Ltd. (Osaka, Japan). Antibodies against occludin, NF-E2-related factor 2 (Nrf2), heme oxygenase 1 (HO1), NAD(P)H quinone dehydrogenase 1 (NQO1), and superoxide dismutase 2 (SOD2) were purchased from Santa Cruz Biotechnology (Santa Cruz, CA, USA), and β-actin was purchased from Cell Signaling Technology (Beverly, MA, USA).

### 2.2. Manufacture of Koji Amazake

**Figure 1:**
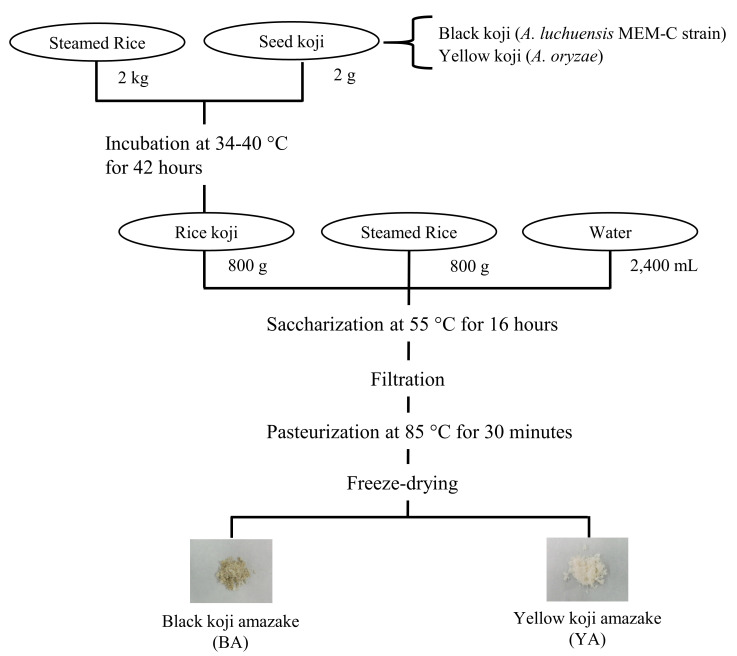
Schemes of Amazake manufacture. The black koji was derived from *A. luchuensis* MEM-C strain and the yellow koji was from *A. oryzae.* Black and yellow rice koji were incubated at 34–40 °C for a total of 42 h, first at 34 °C for 16 h, then at 36 °C for 8 h, and finally at 40 °C for 18 h. Then, 800 g of cooked rice, 800 g of rice koji, and 2400 mL of water were mixed in a 5 L-volumetric flask and saccharified at 55 °C for 16 h. After saccharification, the mixture was filtered and pasteurized at 85 °C for 30 min and finally freeze-dried to collect Amazake powder. BA: black koji Amazake; YA: yellow koji Amazake.

Two kinds of Amazake were made from steamed rice and rice koji (Figure 1). All the rice used in this study was white Japanese rice. A starter called “seed koji” was added at a ratio of 0.1% steamed rice (*w*/*w*). “Seed koji” contained black koji (*Aspergillus luchuensis* MEM-C strain) or yellow koji (*Aspergillus oryzae*), respectively. The black koji was derived from *A. luchuensis* MEM-C strain (Kirishima Shuzo Co., Ltd., Miyazaki, Japan) and yellow koji (Akita Konno store Co., Ltd., Akita, Japan) was from *A. oryzae*. In detail, 2 g of black or yellow seed koji and 2 kg of steamed rice were incubated at 34–40 °C for a total of 42 h, first at 34 °C for 16 h, then at 36 °C for 8 h, and finally at 40 °C for 18 h. Next, 800 g of cooked rice, 800 g of rice koji, and 2400 mL of water were mixed in a 5 L volumetric flask and saccharified at 55 °C for 16 h. After saccharification, the mixture was filtered and pasteurized at 85 °C for 30 min. Finally, koji Amazake was diluted about five-fold with distilled water to a Brix of less than six and freeze-dried to collect Amazake powder. Rice koji and koji Amazake were stored at −20 °C. Yellow koji Amazake (YA) with *A. oryzae* for saccharification is the most common type of Amazake on the Japanese market. In contrast, black koji Amazake (BA) was first manufactured with the *A. luchuensis* MEM-C strain in this study.

### 2.3. Measurement of Sugars Composition in Amazake

The Amazake before lyophilization was centrifuged at 14,500 rpm for five minutes. The supernatant was accordingly diluted by water, and an equal volume of acetonitrile was added. After centrifugation at 14,500 rpm for two minutes, the supernatant was filtered through a 0.45 μm filter and used as the sample for the high-performance liquid chromatography (HPLC) system (SHIMADZU CORPORATION, Kyoto, Japan). For analyzing glucose and maltose, the separation column was Shodex Asahipak NH2P-50 4E (Showa Denko Co., Ltd., Tokyo, Japan), the column oven temperature was 40 °C, the mobile phase was 75% acetonitrile, and the flow rate was 1.0 mL/min. For analyzing isomaltose and isomaltotriose, the separation column was Shodex SUGAR SZ5532 (Showa Denko Co., Ltd.), the temperature of the column oven was 60 °C, the mobile phase was 75% acetonitrile, and the flow rate was 1.0 mL/min. The solution was detected by a differential refraction detector (RID-20A, SHIMADZU CORPORATION).

### 2.4. Measurement of Organic Acids Composition in Amazake

The Amazake before lyophilization was centrifuged at 14,500 rpm for 5 min and the supernatant was injected into the HPLC system after filtration through a 0.45 μm filter. The separation column was Shim-pack SCR-102H (SHIMADZU CORPORATION), the column oven temperature was 45 °C, the mobile phase was five mmol/L *p*-toluenesulfonic acid, the reaction reagent was a 100 μmol/L EDTA-20 mmol/L Bis-Tris, and the flow rate was 0.8 mL/min. The solution was detected by a conductivity detector (CDD-10Avp, SHIMADZU CORPORATION).

### 2.5. Measurement of Ferulic Acid in Amazake

The Amazake before lyophilization was centrifuged at 14,500 rpm for 2 min to obtain the supernatant. The supernatant was then two-fold diluted by 50% ethanol following centrifugation at 14,500 rpm for 2 min. Finally, the supernatant was filtered through a 0.22 μm filter and used as the sample for HPLC. The separation column was Cadenza CD-C18 (Imtakt Co., Ltd., Kyoto, Japan), the column oven temperature was 40 °C, and the mobile phase A was 0.2% formic acid; mobile phase B was acetonitrile. The gradient eluting condition was 98% of A to 60% of A in 0–16 min, and the flow rate was 1.0 mL/min. The solution was detected at 320 nm by a photodiode array detector (SPD-M30A, SHIMADZU CORPORATION).

### 2.6. Measurement of Dietary Fiber in Amazake

Dietary fiber in the Amazake was measured by the Prosky method [16].

### 2.7. Animals Experiment Design

The animal experimental protocol was drafted according to the Animal Care and Use Committee guidelines of Kagoshima University (Permission NO. A12005). Male C57BL/6N mice (five weeks of age) from Japan SLC Inc. (Shizuoka, Japan) were housed separately in cages with wood shavings bedding under controlled light (12 h night/12 h day) and temperature (24 °C) and free access to water and feed. After acclimatization for one week, the mice were randomly divided into five groups and tested for two months (n = 4): normal diet (ND) group, Western diet (WD) group, WD + 10% BA group, and WD + 10% YA group. The ND contained 3% lard and 3% soybean oil; the WD contained 30% lard, 3% soybean oil, and 1% cholesterol. The 10% Amazake in the feed was designed by subtracting 0.3% lard, 0.6% casein, and 9.6% corn starch (Table A1). Normal water was provided for the ND group and 4% fructose water for the WD group. The body weights of the mice were measured every week.

### 2.8. Measurement of Biochemical Indexes in the Animal Experiment

The hepatic mouse monocyte chemotactic protein-1 (MCP-1), serum endotoxin, and fecal immunoglobulin A (IgA) were measured with a kit (Thermo Fisher Scientific Inc., Waltham, MA, USA) according to the manufacturer’s instructions. Fecal mucin content was measured with the Fecal Mucin Assay Kit (Cosmo Bio Co., Ltd., Tokyo, Japan) according to the manufacturer’s instructions. Intestinal occludin protein, Nrf2, HO1, SOD2, and NQO1 were detected by a Western blot assay, as per previous our study [17]. Protein expression was quantified by the densitometry of four Western blot images with LumiVision Analyzer140 (TAITEC Co., Ltd., Saitama, Japan). These data were normalized to the ND group, which was set at 1.0.

### 2.9. Gut Microbiota Analysis by 16S rRNA Gene Sequencing

The feces DNA was extracted by the FastDNA SPIN kit for Feces (MP Bio Japan K. K., Tokyo, Japan). The composition of gut bacterial communities was analyzed by sequencing 16S rRNA genes, as described in our previous paper [17]. The sequences were grouped in amplicon sequence variants (ASVs) with 97% similarity by QIIME 2.0.

### 2.10. Statistical Analysis

Significant differences between groups were determined by Dunnett’s test by GraphPad Prism 9 (GraphPad Software Inc., Boston, MA, USA). Pearson correlations between mouse biochemical indexes and the relative abundance of the gut microbiome were calculated by GraphPad Prism 9. A probability of *p* < 0.05 was considered significant.

## 3. Results

### 3.1. Analysis of Amazake Ingredients

The major ingredients of both BA and YA Amazake are shown in Table 1. Compared to YA, BA Amazake contained higher amounts of ferulic acid, dietary fiber, isomaltose, isomaltotriose, total organic acids, citric acid, pyroglutamic acid, and malic acid. Notably, citric acid comprised approximately 90% of BA’s organic acids and was 20-fold higher than in YA, resulting in a lower pH.

### 3.2. Effects of Amazake on Expressions of Gut Antioxidant Proteins

**Figure 2:**
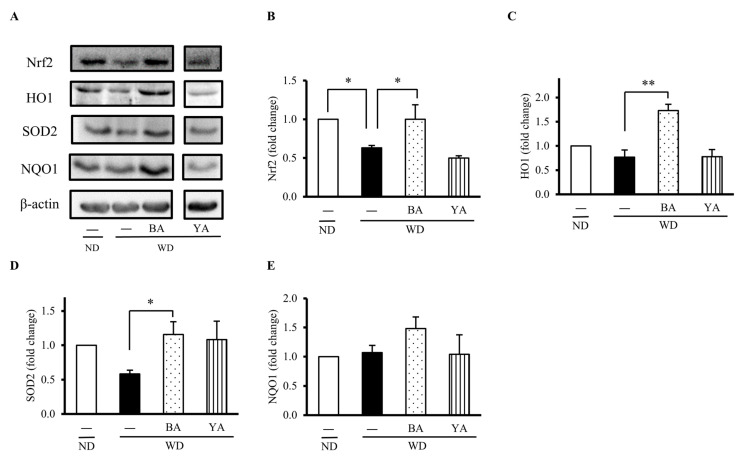
Effects of Amazake on gut antioxidant proteins in the animal experiment. Western blotting images are shown in (**A**) gut Nrf2, HO1, SOD2, and NQO1 protein expressions. Fold changes of (**B**) Nrf2, (**C**) HO1, (**D**) SOD2, and (**E**) NQO1 were calculated from the intensity of (**A**) relative to the ND group and normalized by β-actin intensity. These data represent the mean ± SE for each group. Columns with symbols significantly differ (* *p* < 0.05 and ** *p* < 0.01 versus the WD group by Dunnett’s test). ND: normal diet; WD: Western diet; BA: black koji Amazake; YA: yellow koji Amazake; Nrf2: NF-E2-related factor 2; HO1: heme oxygenase 1; NQO1: NAD(P)H quinone dehydrogenase 1; and SOD2: superoxide dismutase 2.

To explore the effect of Amazake on the gut, we first evaluated gut oxidative status by measuring the levels of some antioxidant factors and enzymes. The typical Western blots for protein detection are shown in Figure 2A. The level of Nrf2, a key transcription factor that regulates the expression of antioxidant proteins, was decreased 0.6-fold in the WD group compared with the ND group and recovered to a normal level by supplementing with BA (Figure 2B). The level of some typic Nrf2 downstream antioxidant enzymes such as HO1 and SOD2 were also significantly recovered from WD-reduced levels by supplementing with BA (Figure 2C,D), but NQO1 was not changed (Figure 2E). On the other hand, these indexes were not recovered in the YA group.

### 3.3. Effects of Amazake on the Expression of Gut Barrier Protein and Inflammation Factors

**Figure 3:**
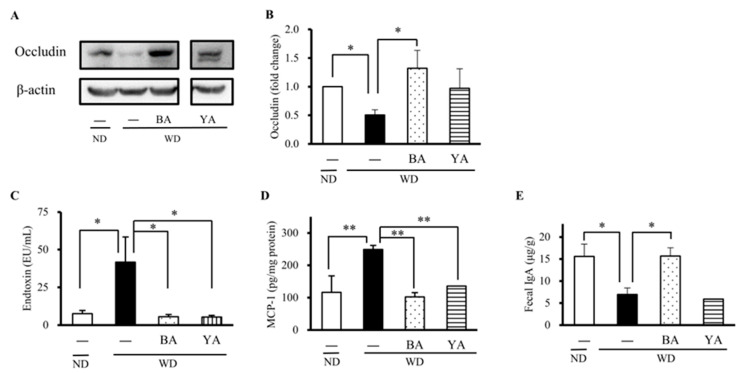
Effects of Amazake on gut barrier function and inflammation in an animal experiment. Western blotting images are shown for (**A**) gut occludin. (**B**) Fold change of occludin calculated from the intensity of (**A**) relative to the ND group and normalized by β-actin intensity. (**C**) Serum endotoxins, (**D**) hepatic MCP-1, and (**E**) fecal IgA. Columns with symbols significantly differ (* *p* < 0.05 and ** *p* < 0.01 versus the WD group by Dunnett’s test). ND: normal diet; WD: Western diet; BA: black koji Amazake; YA: yellow koji Amazake; IgA: immunoglobulin A, MCP-1: monocyte chemotactic protein-1.

Next, we estimated the effects of Amazake ingredients on the gut barrier by investigating the expression of occludin, a physical barrier function. As shown in Figure 3, the level of occludin was significantly decreased in the WD group compared with the ND group and significantly increased in the BA group (Figure 3A,B). It is known that the disruption of the structure and function of the gut barrier allows bacterial toxins and other toxic substances to enter the bloodstream, amplifying systemic inflammation. Thus, we next examined serum endotoxins, which are an indicator of gut barrier function. The results revealed that the WD significantly increased serum endotoxins compared with the ND, and BA and YA significantly decreased them (*p* < 0.01, Figure 3C). Furthermore, the inflammatory cytokine MCP-1 in the liver was significantly increased in the WD group and significantly decreased in the BA and YA groups (Figure 3D). Fecal IgA, a chemical barrier function, was also significantly decreased in the WD group compared with the ND group and significantly increased in the BA group compared with the WD group (Figure 3E).

### 3.4. Effects of Amazake on Gut Microbiota Structure

**Figure 4:**
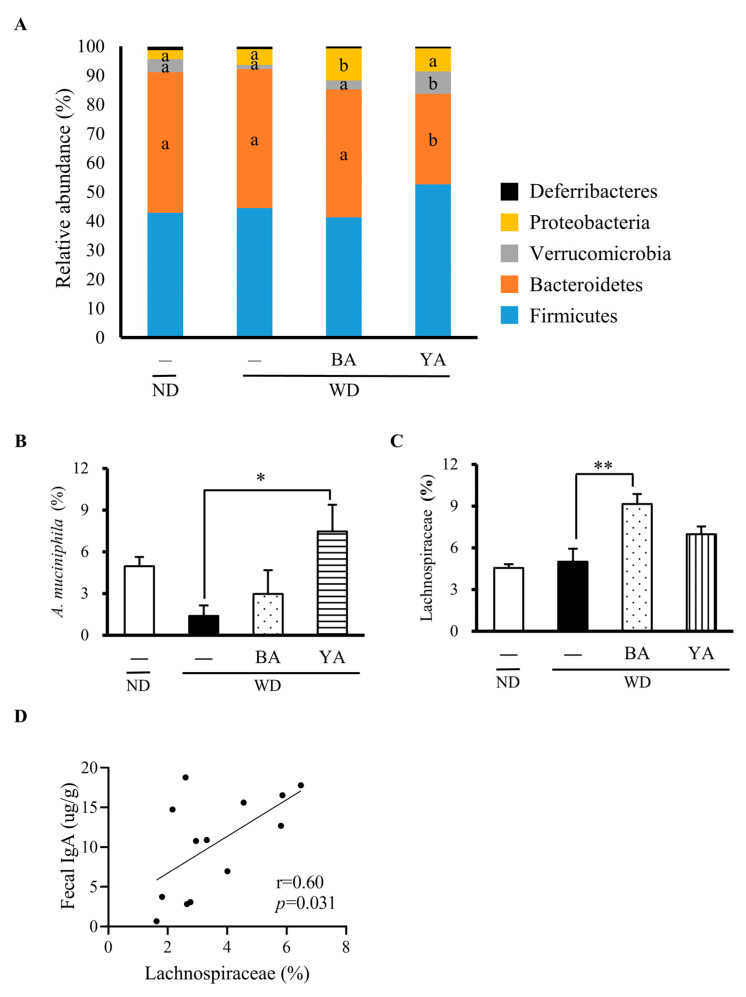
Effects of Amazake on relative abundance at the phylum, family, and species level of gut microbiota. (**A**) Relative abundance of gut microbiota at the phylum level. Relative abundance of (**B**) *Akkermansia muciniphila*, (**C**) *Lachnospiraceae.* (**D**) Pearson correlations between the abundance of *Lachnospiraceae* and the concentration of fecal IgA. These data represent the mean ± SE for each group. Columns with different letters significantly differ (*p* < 0.05). Columns with symbols significantly differ (* *p* < 0.05 and ** *p* < 0.01 versus the WD group by Dunnett’s test). ND: normal diet, WD: Western diet, BA: black koji Amazake, YA: yellow koji Amazake. Columns with different letters significantly changed.

Compared to the WD group, the relative abundance of *Proteobacteria* was increased in the BA group while *Verrucomicrobia* and *Firmicutes* were increased in the YA group (Figure 4A). At the family and species level, *Lachnospiraceae* was increased in the BA group and *A. muciniphila* was increased in YA compared with WD (Figure 4B,C). Next, we performed a Pearson correlation analysis between mice biochemical data and gut microbiome data from the ND, WD, BA, and YA groups. As shown in Figure 4D, a positive correlation (r = 0.60, *p* = 0.031) was observed between the relative abundance of *Lachnospiraceae* and fecal IgA in both the BA and YA groups. However, there was no correlation between *Akkermansia* and mice biochemical data (e.g., MCP-1), although *A. muciniphila* was 5-fold increased in the YA group compared with the WD group.

## 4. Discussion

### 4.1. Difference between the Two Types of Amazake Ingredients

In this study, we first analyzed the major ingredients of two types of Amazake manufactured with different koji (*Aspergillus*), then compared their effects on gut barrier function and gut microbiota. YA with yellow koji (*A. oryzae*) for saccharification is the most common type of Amazake on the Japanese market. In contrast, BA was first manufactured with black koji (*A. luchuensis* MEM-C strain) in this study. Nutrient data show that carbohydrates accounted for approximately 90%, excluding water, of the Amazake, which was almost the same as the market Amazake [18]. However, the BA Amazake showed much higher amounts of other functional ingredients compared to that of YA. It is noted that citric acid in BA Amazake was 20-fold higher than in YA, resulting in a lower pH. The reason why BA has an even higher citric acid content than YA is that the *A. luchuensis* MEM-C strain is an allied species of *A. kawachi,* which has a citrate exporter [19]. *A. luchuensis* is reported to possess ferulic acid esterase, which degrades rice cell wall polysaccharides to ferulic acid [20] and contributes to producing the higher ferulic acid contents of BA.

In addition to the oligosaccharides and organic acids analyzed in this study, Amazake has been reported to be rich in vitamins B2, B3, B7, and B9 [10]. Three weeks of vitamin B2 supplementation caused a reduction in systemic oxidative stress and anti-inflammatory effects in CD patients [21]. The effects of the Amazake production process on the vitamin B complex need to be investigated in a future study.

### 4.2. Effects of Amazake on Gut Barrier Function and Gut Microbiota

**Figure A1:**
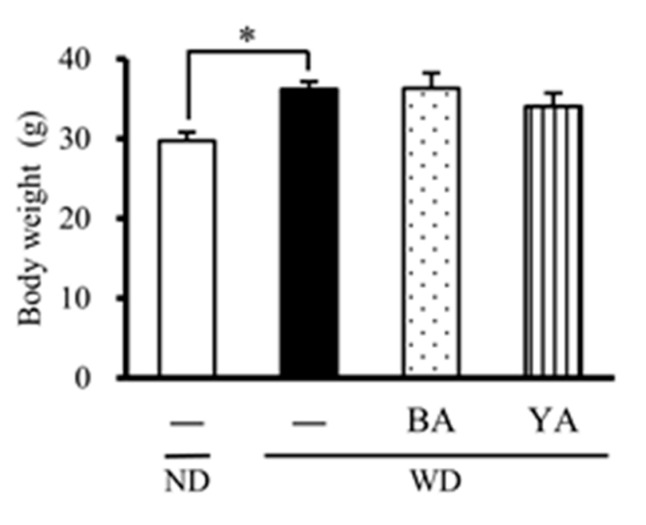
Body weight of each group at the endpoint. The data represent the mean ± SE for each group. Columns with symbols significantly differ (* *p* < 0.05 versus WD group by Dunnett’s test). ND: normal diet; WD: Western diet; BA: black koji Amazake; YA: yellow koji Amazake.

To explore the effects of Amazake on gut barrier function and gut microbiota, a mouse dietary model fed with aWD containing higher fat and fructose for 15 weeks was used to induce gut disturbance [9]. Mice’s body weight significantly increased in the WD group compared to the ND group, and BA and YA did not improve the WD-induced body weight (Figure A1). We chose occludin as the gut barrier marker in the animal experiment because gut occludin was observed to decrease in both human UCand DSS-induced UC mice [22] Occludin is a tight-junction protein acting as a physical barrier. When it was disrupted, the gut barrier allows bacterial toxins and other toxic substances enter the bloodstream (known as serum endotoxins). IgA is the most abundant antibody secreted into the gut and plays a crucial role in immune exclusion by neutralizing pathogenic toxins and preventing pathogen attachment and invasion across the gut barrier [23]. Therefore, we used gut occludin, serum endotoxin, and fecal IgA as indicators of damaged barrier function. Our data revealed that the expressions of gut occludin and fecal IgA were significantly decreased in the WD group, resulting in increased serum endotoxin, MCP-1 (Figure 3), and these events were significantly improved by BA. Therefore, we considered that BA could improve WD-caused gut barrier damage to prevent chronic inflammation.

The concern is how BA improves WD-caused gut barrier damage. It has been reported that Nrf2 working as a master regulator of cellular oxidative levels reduced gut mucosal injury by suppressing oxidative stress and affected gut tight-junction proteins and the apoptosis of cells to regulate intestinal permeability [24]. In this study, BA significantly upregulated the expressions of antioxidant factors and enzymes such as Nfr2, HO1, and SOD-2. At the same time, the expression of gut occludin barrier protein and fecal IgA were also observed to be significantly enhanced by BA. Thus, it is possible that BA enhanced gut antioxidant activities to prevent against WD-caused gut barrier damage. Future study is needed to confirm this correlation between BA antioxidants and gut damage protection in the gut.

It was also noticed that BA is rich in organic acids, notably citric acid, which has been reported to have various effects on the host and bacteria. Citric acid is widely used in foods as an acidifier and pH adjuster and as an antibacterial agent instead of antibiotics for animal feed [25]. These effects were caused by non-dissociated organic acid passing through the bacterial cell membrane and releasing hydrogen ions inside the cell, resulting in a decrease in intracellular pH and the suppression of the enzyme activity involved in bacterial metabolism, thereby exhibiting bactericidal activity [26]. Organic acids, including citric acid, were reported to improve dysbiosis as well as gut barrier function and inflammation in various animals. The administration of 3% citric acid to broiler chicks improved gut morphology, including increasing the number of goblet cells and villus width/crypt depth [27]. Citric acid of 1.5% concentration suppressed the activation of the signaling pathways involved in inflammatory cytokines and increased the gene expression of tight-junction structures such as claudin, occludin, and ZO-1 in the distal intestine of flatfish [28]. The administration of 0.5 to 1.5% citric acid to quail chicks increased *Lactobacillus* and decreased coliform, *E.coli*, and *Salmonella* in a concentration-dependent manner [29]. In our research, BA had the highest citric acid content (approximately 1.5% of the Amazake), suggesting that the improvement of gut barrier function by BA might be due to citric acid action.

Ferulic acid, one of the polyphenols in Amazake [10], increased occludin and *Akkermansia*, activated the Nrf2 pathway, and decreased endotoxins [30,31,32]. Alginate oligosaccharide and chitosan oligosaccharide activated the Nrf2 pathway and upregulated the downstream expression of HO1 and NQO1 in mice [33,34,35]. In this study, BA contained higher ferulic acid and isomaltose, which possibly contributed to activating the Nrf2 pathway-mediated expression of HO1, NQO1, and SOD2.

Ferulic acid and IMOs including isomaltose and isomaltotriose have been reported to increase *A. muciniphila* in mouse experiments [31,36]. In the YA group, *A. muciniphila* was significantly increased, although YA had fewer IMOs than BA. As shown in Table 1, BA contained high contents of ferulic acid, isomaltose, and isomaltotriose, as well as a high content of citric acid, which resulted in a pH 3.8 of BA and might also decrease gut pH. It is known that a pH under 5.6–5.9 inhibits *A. muciniphila* growth [37]. In fact, we also observed that the growth of *A. muciniphila* was inhibited in the monocultures with 125 μg/mL citric acid. Thus, the fact that BA did not increase *A. muciniphila* was due to its high content of citric acid. In contrast, the YA contained little citric acid, thus the growth of *A. muciniphila* was not inhibited.

It has been reported that *A. muciniphila* secretes the Amuc_1100 protein, which induces the anti-inflammatory cytokine interleukin-10 via toll-like receptor 2 [38,39] and improved colitis in mice [40]. In this study, *A. muciniphila* was 5-fold increased in the YA group compared with the WD group, but there was no correlation between *Akkermansia* and MCP-1. Similar results were also reported for Saskatoon berries, in which *Akkermansia* was 5-fold increased and MCP-1 was decreased in the berry administration group, but there was no correlation between the two [41]. Thus, MCP-1 and endotoxins might be regulated by many barrier factors, not only *A. muciniphila*.

IBD may be involved in innate or adaptive immune responses such as the activation and differentiation of T helper 17 cells (Th17) [42]. In addition, interleukin-17 (IL-17), secreted from Th17 cells, can promote the secretion of IgA and a variety of antimicrobial peptides in the gut to accelerate the healing of intestinal mucosal injury, thereby enhancing the gut barrier function [43]. However, the levels of Th17 and IL-17 were not analyzed, so it is necessary to examine gut immune cells to elucidate the mechanism of the improvement of gut barrier function in more detail.

### 4.3. Relationship between Gut Barrier Function and Gut Microbiota

In the intestine, IgA was the most predominant compared to IgM and IgG secreted by B cells, and IgA bound to antigens such as food and microbes, avoiding direct contact with host epithelial cells [44]. In this study, BA increased IgA, which was positively correlated with the abundance of *Lachnospiraceae*. Superantigens expressed on the surface of commensal bacteria of the family *Lachnospiraceae*, such as *R. gnavus,* bound to IgA variable regions and stimulated potent IgA responses in mice [45]. Furthermore, diets containing organic acids, including citric acid, increased *Lachnospiraceae* in broilers [46], complementing the fact that the citric acid in BA increased *Lachnospiraceae* and IgA.

## 5. Conclusions

Black koji Amazake (BA) contained higher amounts of ferulic acid, isomaltooligosaccharides, and citric acid than yellow koji Amazake (YA), and contributed to protecting gut barrier function to reduce endotoxin intrusion and inflammation. In addition, BA increased *Lachnospiraceae*, which positively correlated with fecal IgA, while YA increased *Akkermansia*, which may contribute to protecting gut mucosa. The detained mechanism of gut barrier protection function by Amazake needs further study to clarify.

## Figures and Tables

**Table 1:** Major ingredients in two Amazake (dried weight).

Components	BA	YA
Ferulic acid (mg/100 g)	0.55	0.008
Dietary Fiber (g/100g)	2.3	1.3
Isomaltose (g/100 g)	9.2	5.2
Isomaltotriose (g/100 g)	0.66	0.24
Total organic acids (mg/100 g)	1760	288
Citric acid (mg/100 g)	1553	76
Pyroglutamic acid (mg/100 g)	111	23
Malic acid (mg/100 g)	8.1	1.3
Lactic acid (mg/100 g)	2.7	31.9
Acetic acid (mg/100 g)	2.5	15.2
pH	3.80	6.28

BA: black koji Amazake, YA: yellow koji Amazake.

## Data Availability

Data is contained within the article or Appendix A.

## Appendix A

**Table A1:** Dietary compositions of each group.

Components (%)	ND	WD		
	ー	ー	BA	YA
Lard	3	30	29.7	29.7
Soybean oil	3	3	3	3
Corn starch	43	15	5.9	5.9
Casein	21	21	20.4	20.4
Sucrose	20	20	20	20
Cellulose	5	5	5	5
Mineral Mix	3.5	3.5	3.5	3.5
Vitamin Mix	1	1	1	1
Cholesterol	0	1	1	1
Choline Bitartrate	0.2	0.2	0.2	0.2
Methionine	0.3	0.3	0.3	0.3
Black koji Amazake (BA)			10	
Yellow koji Amazake (YA)				10
Total calories (kcal/100 g)	371	520	523	523

ND: normal diet; WD: Western diet; BA: black koji Amazake; YA: yellow koji Amazake.
